# Facilitating participation in cardiovascular preventive initiatives among people with diabetes: a qualitative study

**DOI:** 10.1186/s12889-021-10172-6

**Published:** 2021-01-22

**Authors:** Marie Dahl, Susanne Friis Søndergaard, Axel Diederichsen, Frans Pouwer, Susanne S. Pedersen, Jens Søndergaard, Jes Lindholt

**Affiliations:** 1 Vascular Research Unit, Department of Surgery, Regional Hospital Central Denmark, Toldbodgade 12, DK-8800 Viborg, Denmark; 2 Department of Clinical Medicine, Aarhus University, Palle Juul-Jensens Blvd. 82, DK-8200 Aarhus N, Denmark; 3 Centre for Research in Clinical Nursing, Regional Hospital Central Denmark/VIA University College, School of Nursing, Viborg, Toldbodgade 12, DK-8800 Viborg, Denmark; 4 Department of Public Health, Nursing, Aarhus University, Bartholins Allé 2, DK-8000 Aarhus C, Denmark; 5 Department of Cardiology, Odense University Hospital, J.B Winsløws vej 4, DK-5000 Odense C, Denmark; 6 Department of Psychology, University of Southern Denmark, Campusvej 55, DK-5230 Odense M, Denmark; 7 STENO Diabetes Centre Odense, Kløvervænget 112, DK-5000 Odense C, Denmark; 8 School of Psychology, Deakin University, Geelong Waterfront Campus, 1 Gheringhap Street, Geelong, Victoria 3220 Australia; 9 Research Unit for General Practice, Department of Public Health, University of Southern Denmark, J.B. Winsløws Vej 9A, DK-5000 Odense C, Denmark; 10 Department of Cardiothoracic and Vascular Surgery, Odense University Hospital, J.B. Winsløv Vej 4, DK-5000 Odense C, Denmark; 11 Elitary Research Centre of Individualised Medicine in Arterial Disease (CIMA), J.B. Winsløv Vej 4, DK-5000 Odense C, Denmark; 12 Cardiovascular Centre of Excellence in Southern Denmark (CAVAC), J.B. Winsløv Vej 4, DK-5000 Odense C, Denmark

**Keywords:** Type 2 diabetes mellitus, Cardiovascular disease, Prevention, Qualitative research

## Abstract

**Background:**

Type 2 diabetes (T2D) is associated with a significantly increased risk of cardiovascular disease (CVD). The **DIA**betic **CA**rdio**VA**scular **S**creening and intervention trial (DIACAVAS) was designed to clarify whether advanced imaging for subclinical atherosclerosis combined with medical treatment is an effective strategy to develop individualised treatment algorithms for Danish men and women with T2D aged 40–60. But in the DIACAVAS pilot study, the uptake was only 41%. Consequently, we explored how people experienced living with T2D to understand how to improve the uptake in initiatives targeting the prevention of CVD.

**Methods:**

We used semi-structured interviews to obtain information on how the respondents experienced having T2D. For supplementary information, we used structured interviews on e.g. socioeconomic factors. From April to October 2019, 17 participants aged 40–60 years were recruited from general practices and diabetes outpatient clinics in Denmark. Several levels of analysis were involved consistent with inductive content analysis.

**Results:**

The participants’ experiences of living with T2D fell along two continuums, from an emotional to a cognitive expression and from reactive to proactive disease management. This led to identification of four archetypal characteristics: *(I)* powerlessness, *(II)* empowerment, *(III)* health literacy, and *(IV)* self-efficacy. These characteristics indicated the importance of using different approaches to facilitate participation in cardiovascular preventive initiatives. Additionally, findings inspired us to develop a model for facilitating participation in future preventive initiatives.

**Conclusion:**

Encouraging people with T2D to participate in cardiovascular preventive initiatives may necessitate a tailored invitation strategy. We propose a model for an invitational process that takes into consideration invitees’ characteristics, including powerlessness, empowerment, health literacy and self-efficacy. This model may enhance participation in such initiatives. However, participation is a general concern, not only in relation to cardiovascular prevention. Our proposed model may be applicable in preventive services for people with T2D in general.

**Supplementary Information:**

The online version contains supplementary material available at 10.1186/s12889-021-10172-6.

## Background

Type 2 diabetes (T2D) is associated with an elevated risk of microvascular complications, such as nephropathy, neuropathy and retinopathy, as well as higher mortality rates due to macrovascular events like myocardial infarction and ischemic stroke [1]. Nevertheless, the most effective strategy to prevent cardiovascular disease (CVD) among people with T2D remains uncertain. Detection of early signs of atherosclerosis may be a solution. Therefore, the **DIA**betic **CA**rdio**VA**scular **S**creening and intervention trial (DIACAVAS) has been designed to clarify whether advanced imaging for subclinical atherosclerosis combined with preventive medical treatment is an effective strategy to develop individualised treatment algorithms among Danish men and women with T2D aged 40–60. However, the uptake in the DIACAVAS pilot study was only 41% [2]. This finding is in line with earlier studies suggesting that persons with T2D are less likely to participate in initiatives such as DIACAVAS. For example, Geppert et al. [3] found that only 44% of participants with diabetes were willing to participate in a hypothetical research study. Additionally, 48% of people with T2D detected through screening did not attend their 12-year follow-up in the Anglo-Danish-Dutch study that explored whether screening for T2D and subsequent intensive treatment could reduce cardiovascular events (ADDITION-study) [4]. Conversely, the uptake was found to be higher in the general populations invited to attend screening for multiple CVD: it ranged from 62 to 74% [5–7] and up to 84% among men invited to attend screening for abdominal aortic aneurysm [8]. However, in a randomised trial offering screening for atrial fibrillation, the uptake was only 48.6% [9]. But in these studies, the uptake was not reported for people with diabetes.

Recent systematic reviews found that non-participation in diabetes outpatient settings were related to appointment logistical issues, such as lack of flexibility in the outpatient clinic and communication failures as well as socioeconomic and psychological factors [10, 11]. Additionally, Lee et al. found that non-participation was associated with younger age, smoking and higher HbA_1c_ [11]. By contrast, in the follow-up assessment of the ADDITION-study in individuals with T2D detected through screening, HbA_1c_ levels were not significantly associated with participation, whereas concurrent high burden of disease defined by Charlson’s comorbidity index score > 2 was significantly associated with lower uptake [4]. In a multicentre randomised study evaluating gender and trial scenarios on willingness to participate in cardiovascular prevention measures, trials among 783 participants (21.4% of the men and 17.8% of the women had diabetes) indicated that women with diabetes were less likely to participate (relative risk (RR): 0.83; 95% confidence interval (CI) 0.56–1.21)), whereas men with diabetes were more likely to participate (RR: 2.21; 95% CI 1.15–4.26) [12].

From an individual’s perspective, a systematic review found reasons for non-participation in diabetes outpatient clinics could be categorised into *I)* poor relationship with the healthcare professionals (HPs), *II)* low perceived benefits of participating and balancing the costs, and *III)* coping strategies like handing over management of diabetes to family members in particular [10]. By contrast, people with T2D who previously participated in diabetes clinical trials expressed both altruism and self-interest as motives for participating [13]. Similarly, Broholm-Joergensen et al. [14] found in an interview study among Danish patients from general practice aged 45–64 that participants with T2D participated in health checks, because they were aware of the importance of managing their risk after experiencing a CVD event. Moreover, participating was a strategy they used to validate a current lifestyle [14].

In cardiovascular screening, especially face-to-face invitations by treatment providers have been found to be effective in improving the uptake [15, 16]. In an analysis of different invitation approaches to NHS health checks within 30 general practices, the uptake of face-to-face invitation was 71.9% compared to 43% for telephone invitation and 29.5% for written invitation [16].

Overall, participation in initiatives targeting the prevention of CVD is a general challenge among people with T2D, but providing evidence of treatment of diabetes relies on successful recruitment to clinical trials and screening examinations. According to the WHO, the uptake of a screening programme must exceed 70% in order to be effective [17], and a poor uptake reflects the presence of potential barriers that must be identified and overcome to ensure its effectiveness. Thus, the DIACAVAS investigators suggested to improve the uptake before conducting a large-scale trial. Consequently, our aim was to use qualitative research methods to explore how people experience living with T2D to understand how to achieve higher uptakes in initiatives targeting the prevention of CVD.

## Methods

### Study design

We conducted a qualitative study with semi-structured and structured interviews.

### Setting

Participants were recruited from general practices and diabetes outpatient clinics from three of the five Regions in Denmark (Central Denmark Region, Region of Southern Denmark and North Denmark Region). The majority of the Danish population (98%) is registered with a general practitioner (GP) [18]. GPs are coordinators of the medical care for the majority of people with T2D, only a minority is monitored in outpatient clinics. Regardless of provider, it is free of charge. All the data were collected within the setting where the participants were recruited and monitored for their T2D.

### Sampling and recruitment

A purposeful sampling strategy was applied by inviting men and women with T2D aged 40–60 from different settings without specific duration of T2D. The focus of our study was to explore the participants’ experiences of living with diabetes and to gain an in-depth understanding of how to facilitate participation in terms of reasons that were not necessarily recognised by the interviewees. Therefore, we recruited participants who had not been invited to an initiative targeting the prevention of CVD and thus had no prior experience deciding whether or not to accept such an invitation.

In total, 24 individuals were approached of whom 17 (70.8%) agreed to participate. “Other things to do / too busy” was stated as the main reason for declining to participate in the study. Recruitment was performed face-to-face either by the participants’ HPs or by the first author. The participants decided the time of their interviews.

### Data collection

Individual face-to-face interviews were conducted from April to October 2019, inspired by Brinkmann and Kvale’s qualitative methodological considerations in relation to conducting an interview study [19]. The interviews lasted 20–45 min, including the time it took obtaining informed consent.

Data were collected by semi-structured interviews combined with structured interviews and notes. As recommended by Brinkmann and Kvale [19], we developed a semi-structured interview guide specifically for this study with references to the literature on peoples’ experiences of living with T2D and health promoting services, including diabetes-related preventive initiatives and cardiovascular preventive initiatives like screening and clinical trials (Additional file 1). The structured interview conformed to the questionnaire used in DIACAVAS for collecting information on cardiovascular symptoms and morbidity, smoking habits, family history of CVD and quality of life (EQ-5D-3L). The DIACAVAS questionnaire was developed in accordance with a literature review on diabetes-specific factors for assessing the individuals’ characteristics including cardiovascular risk [2]. The DIACAVAS questionnaire was supplemented with information on education and employment for the purpose of this study. Notes were made after the interview relating to e.g. important unrecorded statements and interview setting.

Prior to the study, we pilot-tested the interview guide on two participants fulfilling the inclusion criteria of this study. Results of these two pilot interviews did not lead to changes to the interview guide. Thus, we decided to include these interviews in the final interview study. The semi-structured interviews were audio-taped and transcribed verbatim by a research assistant. Data collection was continued until the two researchers responsible for performing the analysis deemed that no further data would add information to the analysis [20].

### Data analysis

We performed an iterative and inductive content analysis following the recommendations by Elo and Kyngas [20]. Two of the authors (MD and SFS) performed the analysis, and started by reading and rereading the interviews to get an impression of the empirical data. Next, participants’ statements were identified and coded, which was a collaborative process that included discussion and interpretation. Then, main categories and subcategories were generated.

In accordance with the identified experiences of living with T2D, we raised the findings to a new analytical level by using a reflexive approach, recognising that qualitative research is part of a larger and unified understanding of the social world [21–23]. Using this approach, we developed a model of participants’ experiences, which subsequently led to the design of another model to facilitate participation in future cardiovascular preventive initiatives. Finally, we performed an additional analysis in which we used data from the structured interviews to identify socioeconomic factors related to the participants’ positions in the model. The analysis process and findings were discussed until all authors were in agreement.

In this analysis, we used the software program NVivo, version 12 Pro (QRS International Pty Ltd., Victory, Australia) as a structural tool to facilitate the analysis.

## Results

In total, 17 respondents participated in the study. Selected results from the structured interviews and notes are listed in Table 1.

**Table 1:** Basic details on participants

Participant	Gender	Age	T2D duration	Working status	Completed level of education
1	Female	50–54	> 10 years	Outside the labour market	Secondary
2	Female	40–44	≥ 10 years	Employed	Secondary
3	Male	50–54	1–4 years	Outside the labour market	Vocational
4	Male	45–49	1–4 years	Employed	Vocational
5	Male	55–60	1–4 years	Employed	Vocational
6	Male	40–44	5–9 years	Employed	Compulsory
7	Female	45–49	5–9 years	Outside the labour market	Vocational
8	Female	55–60	1–4 years	Employed	Tertiary
9	Male	55–60	1–4 years	Outside the labour market	Compulsory
10	Male	55–60	< 1 year	Employed	Tertiary
11	Male	55–60	≥ 10 years	Employed	Secondary
12	Male	50–54	≥ 10 years	Self-employed	Compulsory
13	Male	50–54	≥10 years	Employed	Compulsory
14	Male	50–54	1–4 years	Self-employed	Vocational
15	Male	40–44	< 1 year	Outside the labour market	Compulsory
16	Male	45–49	5–9 years	Self-employed	Secondary
17	Female	40–44	1–4 years	Employed	Vocational

**Fig. 1:**
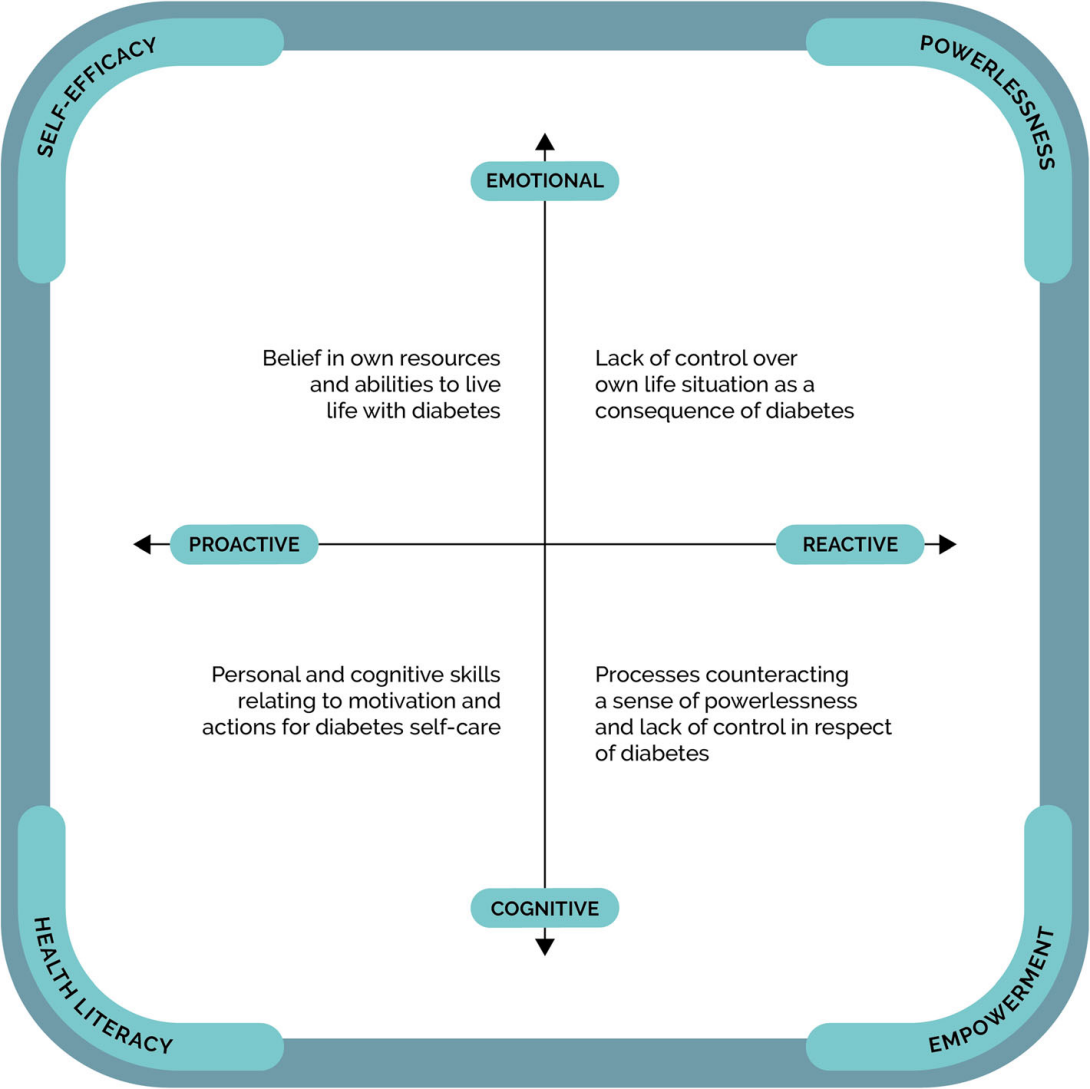
Archetypal characteristics relating to experience of living with type 2 diabetes

In the analysis, we found that the participants’ experiences of living with T2D fell along two continuums from an emotional to a cognitive expression and from reactive to proactive behaviour which reflected how they managed T2D in their daily lives. This led to identification of four archetypal characteristics: *(I)* powerlessness, *(II)* empowerment, *(III)* health literacy, and *(IV)* self-efficacy. We conceptualised these archetypal characteristics in the model below (Fig. 1).

**Fig. 2:**
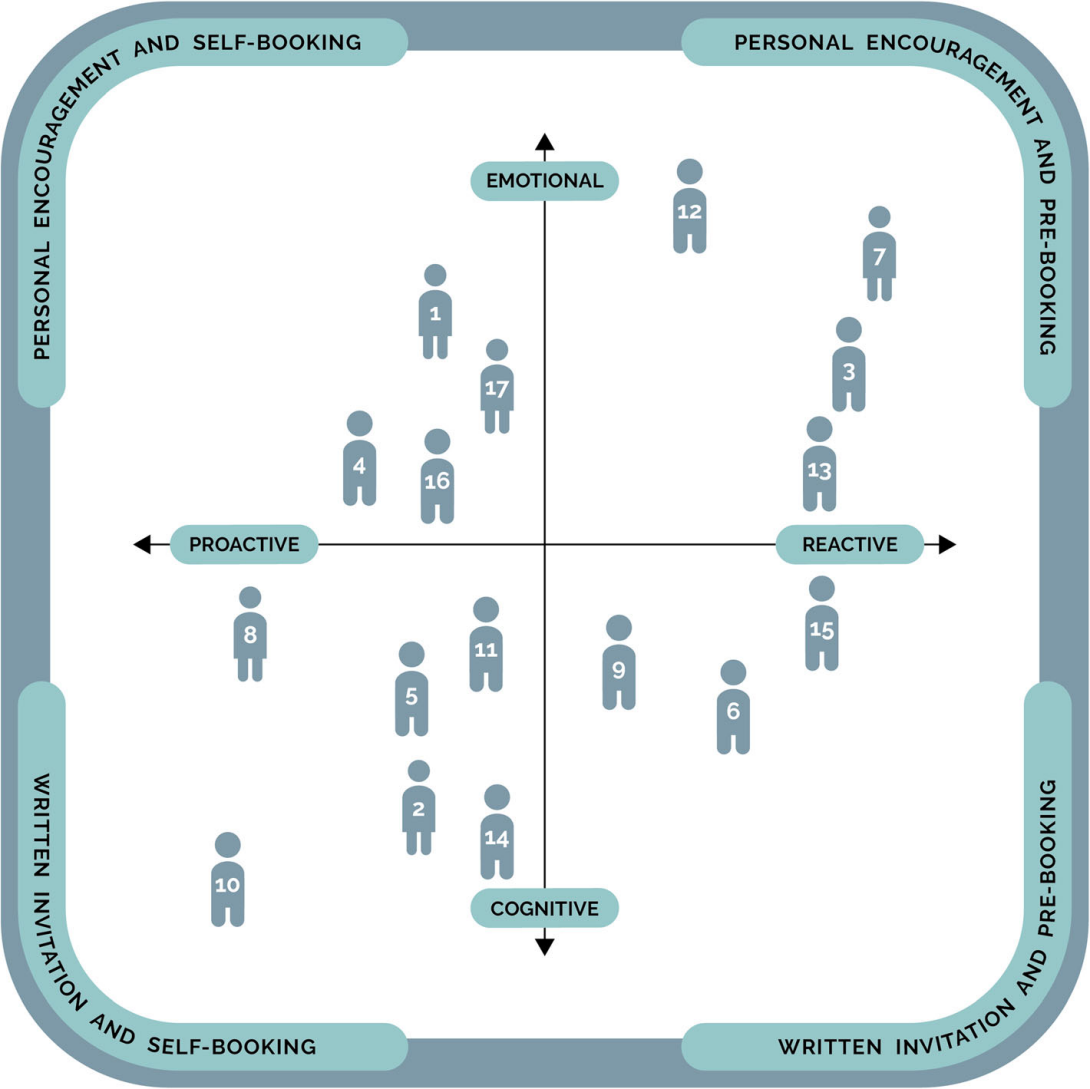
Illustration of invitation strategies for facilitating participation in cardiovascular preventive initiatives

Furthermore, the archetypal characteristics and statements made by our participants indicated the importance of using different invitation approaches to promote participation in preventive initiatives in accordance with their positions in the model (Fig. 2). In Fig. 2, the 17 individuals represent the study participants and the numbers refer to their study numbers listed in Table 1. The participants’ positions in the model are based upon our interpretation of the findings.

### Powerlessness

Participants who displayed the archetypal characteristic of powerlessness expressed how overwhelmed they felt living with T2D. Their statements indicate the predominance of their emotions and a limited ability to undertake diabetes self-care tasks, particularly with respect to following dietary recommendations.

### Conflict between daily life and T2D

Participants in this group dealt with diet management by adopting a few self-selected recommendations and then relapsing to old habits. This happened even though they were aware of the importance of diet in order to prevent or delay diabetes complications, or that their work might be at stake:

*‘You must be careful, you might lose your job; if I push it up to that level, I can stop myself from eating very much for a period of time. It’s a kind of conflict: do you want to live longer or do you not care? … when I’m starting to get it under control, I have a tendency to think that now I deserve a treat’* (Participant 12, male, age group 50–54).

In this way, finding living with diabetes challenging led to a feeling of having lost control of their lives, to personal conflicts and thus, a sense of incapacity.

Suboptimal glycaemic control leads to a range of negative emotions:

*‘When you’ve not improved over a period of time, I wonder what they [HPs] might be thinking: can you not control it? It’s your life and we’re helping you’* (Participant 12, male, age group 50–54).

Thus, suboptimal glycaemic control gives the HPs (and the person with diabetes) the impression that the person with diabetes is “non-adherent”, “not working hard enough”, and *this notion of* insufficient adherence leads to feelings of embarrassment and consequently loss of self-respect:

‘*I’m embarrassed and worried. I do know that the nurses just say: “Well, we must improve on that” … but as a grown-up man, it really can’t be true that I’m not in control of my own life. After all, one should be a role model to the children. That’s how I feel – you lose your pride’* (Participant 12, male, age group 50–54).

In this way, facing HPs was experienced as humiliating, resulting in thoughts of cancelling appointments:

*‘Even though I turn up, it’s sometimes difficult to give an answer as to why the figures [blood glucose] are high, because when you’re writing your diary, but I may not write that down in any great detail: it’s almost like making a confession – like a young child at school with the teachers’* (Participant 12, male, age group 50–54).

Such feelings caused an imbalance between persons with diabetes and HPs that reinforced the feeling of powerlessness.

**Give me back: my life.**

Following the diet was difficult regardless of symptoms due to high blood glucose. One participant said:

*‘Then we’re going out for soft ice and the children have a large one and I’m having a small one. Well, it’s true of course that it tastes just as nice; after all, it’s not the size that determines whether you think it’s tasty or not ... but I feel it afterwards, as I get such a headache – my blood sugar is too high and I find that really irritating; it hampers me.’* (Participant 7, female, age group 45–49).

Eating like they used to before the diabetes diagnosis was central to their experience of enjoyment and their sense of control of their own lives. Consequently, the diagnosis influenced the participants’ quality of life.

Another characteristic of participants in this group was that relatives were crucial to diet management. As one expressed:

*‘During Easter, we were having liquorice Easter eggs and when I had had four, my youngest said “Mum, you’re not having any more” and then he moved the bowl … because he knows that I find it difficult to stop. I find that bloody annoying, because I might have liked to have number five ... but my blood sugar had probably already risen dramatically after I’d eaten the third one.’* (Participant 7, female, age group 45–49).

When interference from family was not appreciated, it exacerbated the feeling of losing control and loss of autonomy. In general, the families were co-responsible for managing diabetes in either a negative or positive way for the participants.

Overall, we found that diabetes had a pervasive influence on the participants’ daily lives and caused inner conflicts due to disharmony between desire and diet recommendations that were considered to be restrictions. For this group of participants, the management of diabetes was characterised by powerlessness resulting in lack of control over their living conditions, reflecting that their autonomy was threatened due to their diagnosis of diabetes.

### Facilitating participation for people expressing powerlessness

For participants whose dominant archetypal characteristic is powerlessness, we found that the approach to facilitate participation in preventive initiatives required personal encouragement:

*‘My GP will probably tell me, if I should participate’* (Participant 4, male, age group 45–49).

In accordance with the findings illustrated in our model, we suggest that taking action for this group – for instance by automated systematic electronic pre-booking in terms of providing a scheduled time for the screening examination in the screening invitation or if their GPs recommended participation and booked on their behalf – would increase the likelihood of participation due to their reactive approach to diabetes self-care.

### Empowerment

The findings showed that this group of participants expressed a sense of empowerment in how they handled living with T2D. Their experiences were predominately cognitively controlled but they took a reactive approach to self-care, particularly in relation to diet and exercise recommendations.

### Cognitive assimilation

The participants were quite lenient on themselves, if their tests showed high blood glucose levels:

*‘It’s bloody annoying...but there’ll be ups and downs when you have diabetes. At one appointment, the figures look fine and then at the next, it’s just been Christmas and then the figures are too high; I think you just have to accept it’* (Participant 6, male, age group 40–44).

With this management approach, the participants re-established control over their lives and they managed to maintain this attitude even if they were aware of potential complications due to diabetes:

*‘I sell diabetes lottery tickets and on these, I provide information on the complications of diabetes. Cancer may well be a cruel disease, but diabetes can be just as vicious, and while cancer is one disease, diabetes is six or seven diseases, affecting the eyes, ears, nose, lungs, stomach, intestines and stents for one thing and another’* (Participant 9, male, age group 55–60).

Thus, knowledge per se does not necessarily promote disease management.

### Learning to live with T2D

This lax attitude was also reflected in their perceptions of diabetes and disease management. One participant said:

*‘You don’t have to be more ill than other people just because you have diabetes … you probably shouldn’t be thinking too much about it, because then it becomes an even greater problem than it is. Obviously, you have to think about what you eat, how much and things like that, but still you must concentrate on living every day’* (Participant 6, male, age group 40–44).

Participants expressing such attitudes followed diet recommendations as and when they could without getting a guilty conscience, a strategy they used to prevent diabetes from controlling their lives.

Overall, for these participants, management of diabetes was characterised by empowerment in terms of cognitive reflections, based on insights and understanding of the disease that did not necessarily result in following all recommendations. This approach to diabetes management was acceptable to them, relying on

*‘I try to follow it the best I can’* (Participant 9, male, age group 55–60).

In this way, they re-established control over their lives and their autonomy after having the diabetes diagnosis. Moreover, this approach prevented the potential distress of not following the recommendations fully.

### Facilitating participation for people expressing empowerment

We suggest that a written invitation will encourage participation in preventive initiatives for this type of individual. A pre-booked time might further increase the likelihood of participation due to their reactive behaviour.

### Health literacy

This group of participants demonstrated health literacy by their personal and cognitive competences in relation to motivation and actions that were health-promoting in terms of all parameters of living with T2D.

They faced realities and acted on the basis of these realities:

*‘I thought, you’ll need to make your own treatment plan .... food, exercise, music and massage. I simply created a programme where I walked more and more every day and I weighed one kilo of vegetables which I ate as the day progressed’* (Participant 8, female, age group 55–60).

Similarly, participant 10 said:

*‘I quickly realised that it was all about getting control of it [diabetes] ... it’s up to you to take charge, that’s all there is to it.’* (Participant 10, male, age group 55–60).

In this way, they experienced being in control, which also occurred by their re-established self-perceptions:

‘*When I started walking at the fjord, then I thought that I’d recovered*’ (Participant 8, female, age group 55–60)*.*

Thereby, they maintained their identity.

Several strategies were used to facilitate diabetes self-care, e.g. knowledge and involvement:

*‘I think that we work it out together – it’s a partnership’* (Participant 8, female, age group 55–60).

In this way, shared decision-making produced the sense of maintaining autonomy. Moreover, they were aware whose advice to seek on managing diabetes:

*‘I know people with diabetes and some of them I wouldn’t dream of copying, because I don’t think that they’re looking after themselves very well … but I know one person I need to have a chat with’* (Participant 10, male, age group 55–60).

Diabetes check-ups were also significant for maintaining optimal self-care:

*‘It’s a wake-up call for me. If the figures are high – oops – you’ve been lazy and will have to pull your socks up’* (Participant 11, male, age group 55–60).

However, at festive occasions, some preferred getting the same food as the other guests instead of sticking to the recommended diet:

*‘I want the same food as the others. They’ve got that now, so they treat me as if I didn’t have it. That suits me best’* (Participant 11, male, age group 55–60).

In this way, not being perceived as a disease in social contexts was favoured over optimal diabetes self-care behaviours, but that decision came without a guilty conscience.

Overall, this group of participants managed their diabetes with critical reflection and by being proactive. Thereby, their autonomy remained intact, and being perceived as a person and not an illness was important.

### Facilitating attendance for people expressing health literacy

We suggest that participants displaying the archetypal characteristic of health literacy would be likely to respond to written invitations that offer self-booking in accordance with our model.

### Self-efficacy

We found that this group of participants was determined to manage their T2D. However, to experience management worthwhile, they needed acknowledgement for their efforts from family members and HPs:

*‘I’ve got the same feeling every time, I want to achieve a good result [blood sugar values]; I feel it’s like sitting an exam’* (Participant 16, male, age group 45–49).

In this way, presenting acceptable glucose levels confirmed having been sufficiently “adherent” and thereby “good”. Moreover, being seen by the HPs was considered valuable:

*‘She [GP] tailors her advice to me ... she knows me and is aware of the issues that I have with the various things’* (Participant 17, female, age group 40–44).

Diabetes affected the entire family, and the family members reacted in a supportive way:

*‘My son’s looking after his mother; he’s taken it onboard ... if he’s shopping, he won’t buy white rice or ordinary pasta ... and he’s also the one who’s saying “oh, you know we shouldn’t get that” if I feel like buying something unhealthy’* (Participant 17, female, age group 40–44).

In this way, family members accepted being co-responsible for the diabetes management.

Being diagnosed with diabetes threatened their identity, but they were able to overcome it by seeing themselves as a person rather than a disease. However, diabetes continued to have a strong presence in their daily lives in different ways:

*‘It’s diabetes that’s setting the rules and then you’ll have to get everything else to fit in around living with diabetes ... it’s diabetes deciding the agenda when I’m shopping in the refrigerated section and at the fruit and veg … If I didn’t have diabetes, I’m also not sure that I’d be using my bike quite as much’* (Participant 16, male, age group 45–49).

The same goes for thoughts about complications. Although, facing diabetes complications was stressful, it could also be a motivating factor for self-care:

*‘If you look around the waiting area, you almost feel like leaving straight away, because there are some people where it’s very obvious that they’ve not been in control of things, and it’s food for thought: I really don’t want to end up like that … you really have to take it seriously, because otherwise it’ll come back to bite you. The complications of diabetes are like dark clouds hanging over you, if you’re not careful’* (Participant 16, male, age group 45–49).

By accepting the diagnosis and relying upon managing diabetes properly to prevent complications, they re-established control over their lives after having the diagnosis.

Overall, this group of participants demonstrated self-efficacy, reflecting their belief in own resources and ability to manage life with diabetes.

### Facilitating participation for people expressing self-efficacy

As these participants belong to the emotional part of the model, we suggest that personal encouragement by HPs may increase the likelihood of participation.

### Selecting invitation strategy according to archetypal characteristic

**Fig. 3:**
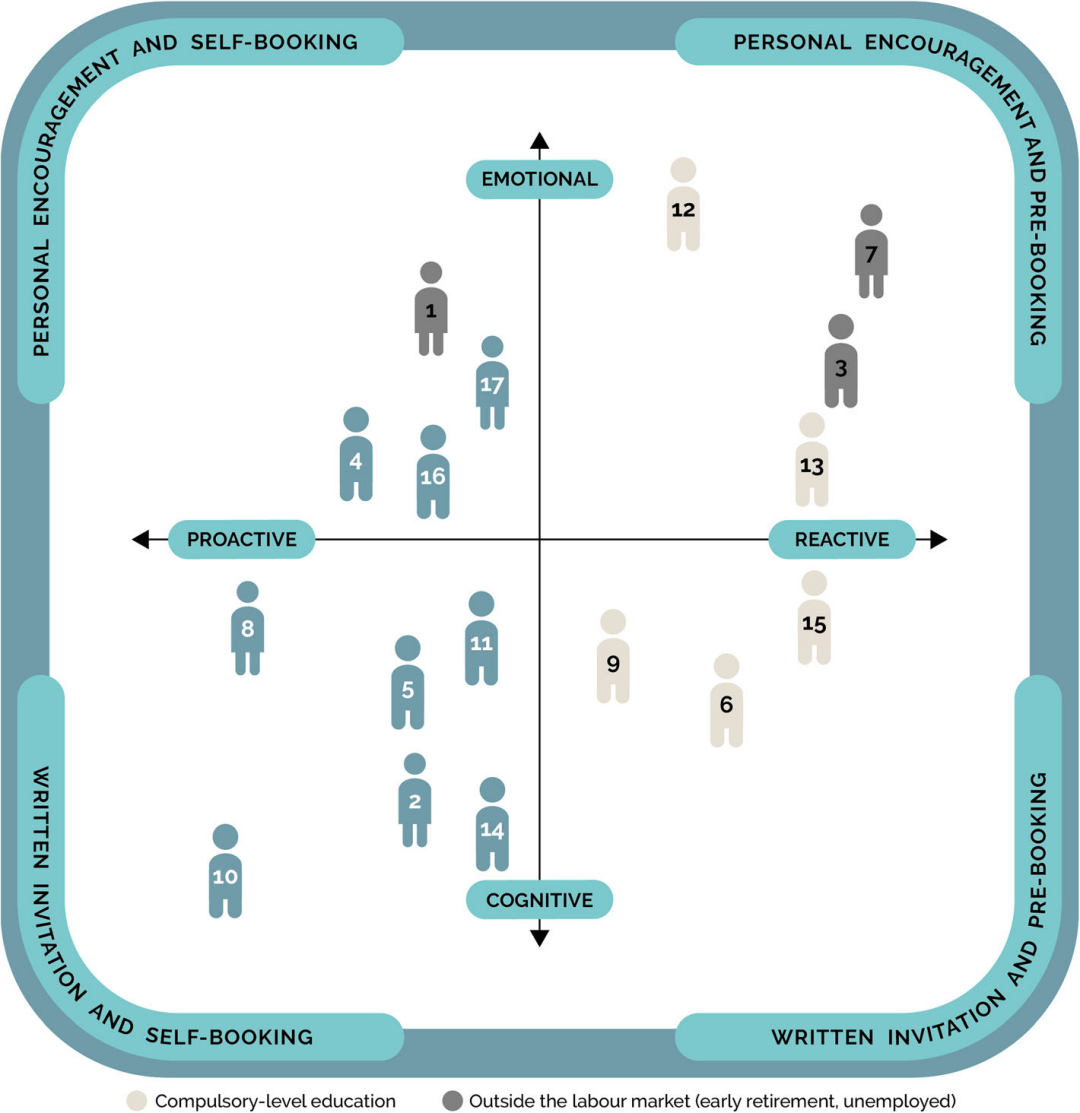
Identification of individuals who need attention prior to participating in cardiovascular preventive initiatives

We performed an additional analysis and found that by comparing the positions of the participants in the model and socioeconomic variables like education and being outside the labour market, it seemed possible to identify the majority of individuals who needed attention prior to being invited to participate in preventive initiatives (Fig. 3). In Fig. 3, the numbers correspond to the participants’ numbers in Table 1, whereas compulsory education and being outside the labour market are indicated by the colours of the “individuals” in the model.

## Discussion

In this study, we examined people’s experiences of living with T2D to understand how to improve the uptake of initiatives targeting the prevention of CVD. We found that the experiences of living with T2D fell along two continuums from an emotional to a cognitive expression and from reactive to proactive disease management. Where the vertical and the horizontal continuums intersected, we identified four archetypal characteristics: *(I)* powerlessness, *(II)* empowerment, *(III)* health literacy, and *(IV)* self-efficacy. Taking these into account seemed important for facilitating participation in CVD preventive initiatives.

We found that the participants expressed one of four dominant archetypal characteristics and their experiences of living with T2D varied enormously both psychologically and in terms of their behaviour. Participants whose dominant archetypal characteristic was powerlessness tended to express quite negative emotions and attitudes as well as the feeling of having lost the vast majority of the control that they used to have of their lives. Overall, they experienced T2D as a threat to their existence and self-esteem. A feeling that influenced their ability to undertake disease self-care and thereby affected their behaviour. In contrast, participants whose dominant archetypal characteristic was self-efficacy expressed positive emotions and attitudes as well as the feeling of being in control of their lives despite their diabetes diagnosis. They believed in their own capacity and skills to live life with T2D, while they needed support and encouragement to manage their disease. As such, we found that the psychological aspects were central to their experience of living with T2D. By contrast, participants whose dominant archetypal characteristic was either empowerment or health literacy expressed a more cognitive attitude towards diabetes self-care. While participants whose dominant archetypal characteristic was health literacy demonstrated health-promoting disease management interpreted in terms of all parameters of living with T2D, the participants for whom empowerment was the dominant characteristic demonstrated that knowledge per see does not prevent individuals from taking a reactive approach to disease management in some parameters. The common denominator for these two types of participants was that they took a cognitive approach to living with T2D, they felt in control of their lives regardless of having diabetes and believed in their own capacity to live life with T2D. Our findings of these diverse experiences of living with diabetes are supported by a recent review by Stuckey and Peyrot [24].

### Tailoring invitation strategies according to archetypal characteristics

In the following, we discuss the possible consequences of these archetypal characteristics in terms of participating in preventive initiatives as well as how our suggested invitation strategies for facilitating participation are supported by existing evidence on persons with T2D.

### Powerlessness

For the group of participants expressing powerlessness, we found personal encouragement to be important for them to accept an invitation to a preventive initiative. Thus, to achieve an acceptable uptake, it is important to collaborate with HPs within diabetes care. Broholm-Joergensen et al. [25] found that GPs’ strategies for retaining individuals in preventive health check programmes constituted a balancing act between trust and power in terms of respect for the individual’s autonomy. Similarly, we found that being seen by HPs for individualised recommendations was important for the participants in the emotional part of the model to facilitate self-care and accepting preventive initiatives.

We think that experiencing feelings of powerlessness could lead to non-participation in preventive initiatives. Similarly, Kibbey et al. [26] found that disempowerment was related to non-participation among people with type 1 diabetes aged 18–30. To facilitate participation in preventive initiatives among people expressing powerlessness, awareness of diabetes-related distress seems crucial. In a systematic review and meta-analysis, Perrin et al. [27] found that diabetes-specific emotional distress (DSD) was prevalent in 36% of people with T2D. DSD refers to psychological distress specific to living with diabetes encompassing a variety of emotions, such as feeling overwhelmed by the demands of self-management, worrying and ruminating about complications, and/or harbouring feelings of guilt or shame, particularly in relation to lifestyle [28, 29]. We found that these feelings were present when our participants expressed powerlessness. This may explain why we found that these participants seemed stuck feeling powerless. Moreover, DSD negatively affects diabetes through reduced self-care [30, 31]. Furthermore, a review by Schram et al. [32] showed that persons with depressive symptoms had a much lower quality of life due to diabetes, and conclusively the authors suggested to screen individuals for depression in diabetes care settings. This is supported by Perrin et al. [33] who found that DSD can be reduced significantly. By reducing DSD and thereby fostering self-care among persons expressing powerlessness, participation in preventive initiatives may also increase, particularly if this group of individuals are invited in accordance with our model, namely by personal encouragement and offering pre-booked appointments.

### Empowerment

Participants with this archetypal characteristic expressed empowerment by taking a cognitive approach, and found motivation and capacity to take control of their disease. Regardless of our interpretation that they are reactive in managing their disease, we found that they were motivated for self-care; they relied on doing what they were capable of in relation to diabetes self-care. Through empowerment, they re-established their autonomy. In interviews with persons with T2D, Boyle et al. [34] found that regardless of being made aware of preventive recommendations, some people decided not to follow them all. Similarly, our findings indicated that knowledge per se does not necessarily promote disease management, even though the participants understood the possible consequences of their decisions. In this way, they acted in accordance with their archetypal characteristic of empowerment [35].

The basis of empowerment is the capacity to think critically and make informed decisions [35]. However, it is questionable whether an invitation would capture the invitees’ attention. Boyle et al. [34] found that persons with T2D reported that they received too much written materials that they did not necessarily read. Therefore, efforts to design an eye-catching invitation would be a prerequisite for ensuring an informed decision and increasing the uptake of preventive initiatives. Additionally, pre-booked appointments may encourage this group of individuals to participate due to their reactive approach to diabetes self-care.

### Health literacy

Individuals who are characterised by being health literate take a cognitive and proactive approach to their diabetes self-care. We found that it was imperative for this type of individual to be in control of their lives and involved in treatment decisions, thus maintaining their autonomy. Similarly, Broholm-Joergensen et al. [25] found that GPs showed respect for their patients’ autonomy and the mutual exchange of views was used as a strategy for keeping them in preventive health check programmes. This could be achieved by sharing in the decision-making process which supports the individual’s decision-making based on informed preferences in collaboration with their HPs. Shared decision-making allows the individual and HPs to be experts and to select treatments that take into consideration the individual’s preferences, values and personal contexts, such as job situations and previous unpleasant experiences of hypoglycaemia [36].

Based on our model, this type of individual seemed the most likely to participate in preventive initiatives. Fisher et al. [37] found that higher education and shorter duration of T2D predicted better recruitment in the non-interventional arm in a randomised trial for facilitating recruitment and retention in clinical trials. We found a similar tendency but with variation in both durations of T2D and education as the job itself also played an important role.

### Self-efficacy

For participants whose dominant archetypal characteristic was self-efficacy, their diabetes self-care gave rise to emotions, regardless of whether they displayed proactive behaviour. Despite their emotions, these individuals believed in their own capability to control diabetes. However, we also found that being seen as an individual by HPs was of importance for their diabetes self-care. Nicolucci et al. [38] found in a multi-country survey among 8596 persons with diabetes (84% had T2D; aged 48–65) that 85.5% reported that their HPs were supportive. However, the survey also indicated insufficient attention to the psychological aspects of living with diabetes, as only 23.7% of the respondents reported that the HPs had asked how diabetes impacted their lives. In addition, Kibbey et al. [26] found that lack of recognition as an individual engaging with diabetes care could cause non-participation in diabetes check-ups. Furthermore, studies have found that individuals with low self-efficacy were less likely to participate in general health checks offered by GPs [39, 40]. By contrast, in a cross-sectional analysis of participation in a study aimed to prevent T2D, CVD and chronic obstructive pulmonary disease, Larsen et al. [41] found that lower self-efficacy was associated with a higher likelihood of getting health checks. We found that self-efficacy per se did not necessarily facilitate participation according to our model, as personal encouragement is also needed.

According to the social cognitive theory, self-efficacy includes confidence in employing the skills necessary to resist temptations, cope with stress and mobilise the resources required to meet situational demands [42]. In terms of having general self-efficacy [42], this type of individual would be capable of making an informed decision as to whether or not to participate when invited for preventive initiatives. However, we found that this type of individual valued personal relations in their diabetes self-care. Therefore, in accordance with our model, we suggest that personalised encouragement would facilitate participation in future preventive initiatives.

### Improving the basis for increasing participation in preventive initiatives

In the DIACAVAS pilot study, the invitation strategy was to send the invitation to a digital mailbox provided by the Danish public authorities without a pre-booked appointment [2]. This digital invitation was chosen as it is an easy and well-known strategy to reach the majority of the Danish population in a safe, secure and inexpensive way [43]. However, 8.2% of the Danish population does not receive digital post due to, for example, language difficulties and disabilities [43]. Hence, invitees without digital mailboxes received the invitation by standard physical post. However, this invitation strategy might have been a contributing factor to the low uptake in DIACAVAS. Larsen et al. [44] found that among those treated for diabetes, CVD or chronic obstructive pulmonary disease, recruitment by digital invitations was lower (Incidence rate ratio: 1.02; 95% CI 1.00–1.04). Norman et al. [45] found that among those offered a pre-booked appointment for a health check within general practice, the uptake doubled compared to those offered an open invitation (59.2 vs. 26.5%).

A theory-based communication strategy (the AASAP – Anticipate, Acknowledge, Standardise, Accept, Plan) proved to be effective in increasing recruitment and retention of individuals with T2D in a clinical trial [37]. The AASAP approach involves verbalising and normalising individuals’ concerns in a non-judgmental way, enabling them to come to their own realistic decisions about what is best for them and their diabetes [37]. This confirms our finding that it is important to involve HPs in future preventive initiatives in order to facilitate participation. Similarly, a review suggested that involving GPs might facilitate screening efficiency and uptake [46]. The AASAP approach may also reduce the likelihood that individuals with T2D experience diabetes-related stigma when HPs discuss the potential relevance of participating in preventive initiatives. Browne et al. [47] found in an interview study among individuals with T2D that experiencing stigma had psychological and behavioural consequences on self-efficacy and self-care. Moreover, experiencing judgmental attitudes from HPs also resulted in subsequently refraining from seeking advice from HPs [47]. Therefore, involving HPs in recruiting people with T2D in CVD preventive initiatives might not necessarily facilitate participation.

Besides facilitating participation when sending out invitations to preventive initiatives in accordance with our model, this invitation approach may also help invitees making an informed decision on whether or not to participate. Dahl et al. [48] found it doubtful that women’s decision not to participate in cardiovascular screening was based on an informed decision. In addition, these non-participating women did not discuss their invitation with their HPs. This underlines the fact that HPs may need to take the initiative to discuss invitations to preventive initiatives.

We found that different invitation approaches are appropriate to facilitate participation in future preventive initiatives. Interventions to improve the uptake in screening and health checks generally have received considerable attention over the last few decades. In a review from 2000, it was concluded that pre-booked appointments and invitations by telephone were likely to be effective in increasing the uptake in screening for mainly cancer, but some studies show the same applies to screening for hypertension and dyslipidaemia and one study concluded it also applied to diabetes [49]. A recent systematic review from 2020 by Bunten and co-workers reviewing invitation methods and the impact of interventions used in NHS health checks supports this notion. They found that written invitations were less effective than telephone or opportunistic face-to-face invitations [50]. The authors concluded that further research would be needed to examine how to enhance existing invitation methods to facilitate the uptake by taking into account especially ethnicity and gender. However, these reviews did not provide any effective strategies to improve the uptake in relation to people with diabetes. Similarly, a recent systematic review of recruitment strategies in diabetes prevention programmes concluded that it was difficult to distinguish any trends in relation to recruitment methods and uptake [51]. Our study contributes with new understanding as to the reasons why tailored invitation strategies are required and suggests ways of tailoring invitations. Likewise, in a recent review on barriers and facilitators to participation in health checks for cardiometabolic diseases offered by GPs, the authors emphasised that it is impossible to develop a one-fits-all invitation strategy [52]. Patient and public involvement (PPI) is a recommended approach to design attractive healthcare services [53] and may also be useful in identifying outcomes of preventive initiatives which are relevant to the invitees. In a recent review incorporating both qualitative and quantitative studies, Gorst et al. [54] found a discrepancy between patient-derived outcomes and those identified in a systematic review of clinical trials. This emphasises the importance of incorporating PPI in designing preventive initiatives to ensure that the initiatives are meaningful to the invitees in order to maximise the uptake. Alongside this interview study, we included PPI with the aim to design an attractive future initiative targeting the prevention of CVD. The findings will be presented in an upcoming article.

A tailored invitation in line with our recommendations necessitates information about the individual’s socio-economic status and experience of living with T2D. But it is not entirely straightforward for researchers or healthcare services to collect the information required for making a tailored invitation strategy. In Denmark, information on education and employment is not included in medical records, but it is readily available from the Integrated Database for Labour Market Research (IDA) [55]. The challenge is to collect the required information on individuals’ experiences of living with T2D. Reviews have suggested to collect such information in diabetes care settings in connection with screening for DSD and depression [27, 32, 56]. A useful tool could be the 17-item Diabetes Distress Scale, which is available in many languages [57]. However, both emotional and cognitive dimensions are important in accordance with our model, and for evaluating these dimensions, the brief nine-item Illness Perception Questionnaire is very useful [58]. Such information could also contribute to tailoring invitations to already implemented preventive initiatives and generally promote diabetes care.

In Denmark, psychometric instruments could be integrated in the national platform for people with diabetes and their GPs. In this way, general practices could help collecting the information required to make tailored invitations. This digital platform was developed as a collaborative project with representatives from the Danish Ministry of Health and Elderly Care and the Danish Organisation of General Practitioners. However, in order to email tailored invitations automatically, the information must be exported to a system that can customise invitations in accordance with national laws on handling personal and sensitive data, such as the web-based software platform Research Electronic Data Capture, REDCap® [59].

### Limitations and strengths

The strength of qualitative research is reaching a new understanding of a phenomenon, including suggestions for practice rather than achieving generalisability [20], which requires testing the results in a randomised setup. Interestingly, the uptake in the DIACAVAS pilot study (41%) [2] corresponds to the number of interviewees who would be likely to respond to the invitation strategy used in DIACAVAS (a written invitation with self-booking) in accordance with our model (35%). This supports our findings of the benefits of using a differentiated invitation strategy. However, our model of facilitating participation needs to be validated in randomised trials, before it may be used in future preventive screening services targeting the prevention of CVD. Additionally, the psychometric instrument for obtaining the required information for implementing a tailored invitation strategy in accordance with our model also needs to be validated.

We did not use validated psychometric instruments to measure the participants’ degrees of powerlessness, empowerment, health literacy and self-efficacy; this could have strengthened our interpretations of the empirical data. Whether it would have strengthened the applicability of our developed model needs further clarification.

This study provides an in-depth understanding of the motives behind disease management and of the importance of tailoring interventions to invitees’ needs and preferences. However, needs and preferences among people with T2D may be more nuanced and individual than conceptualised in the identified archetypal characteristics. Moreover, the suggestions for making tailored invitations to increase the uptake in preventive initiatives are based on our interpretations. Therefore, our findings should be considered preliminary and examined in further studies.

The study population consisted of twelve men and five women. This study explored experiences of living with T2D generally, rather than gender-specific differences. However, it would be interesting to explore possible gender differences, a potential area for future research.

## Conclusion

We conclude that people’s experiences of living with T2D fall along two continuums, from an emotional to a cognitive expression and from reactive to proactive disease management. Where the vertical and the horizontal continuums intersected, we identified four archetypal characteristics. We found that a tailored invitation strategy taking account of these archetypal characteristics may facilitate participation in cardiovascular preventive services among people with T2D. We propose a model for an invitational process that takes into consideration invitees’ characteristics, including powerlessness, empowerment, health literacy, and self-efficacy. Participation is a general concern, not only in relation to cardiovascular prevention. Our proposed model might be applicable in other preventive services for people with T2D.

## Supplementary Information

**Additional file 1.** Interview guide

## Data Availability

The entire transcribed interviews used in this study are not publicly available. But minor anonymised parts are available from the corresponding author upon a reasonable request. This is to protect and maintain participants’ anonymity and confidentiality.

## Abbreviations

ADDITION: Anglo-Danish-Dutch Study of Intensive Treatment In People with Screen Detected Diabetes in Primary Care
AASAP: Anticipate, Acknowledge, Standardise, Accept, Plan
CI: Confidence Interval
DIACAVS: DIAbetic CArdioVAscular Screening and Intervention trial
DSD: Diabetes-specific emotional distress
CVD: Cardiovascular disease
GPs: General practitioners
HbA_1c_: Glycated haemoglobin
HPs: Healthcare professionals
PPI: Participant and Public Involvement
T2D: Type 2 diabetes
