# Supplementary material for: Facilitating participation in cardiovascular preventive initiatives among people with diabetes: a qualitative study
Source: BMC Public Health. 2021 Jan 22;21:203. doi: 10.1186/s12889-021-10172-6 (PMC7824926; doi:10.1186/s12889-021-10172-6)
Supplement: Supplementary file 1 — Additional file 1. Interview guide [file 12889_2021_10172_MOESM1_ESM.docx]

**Additional file 1. Interview guide**

Would you tell me a little bit about yourself?

How did you experience being diagnosed with diabetes?

How do you experience living with diabetes day to day?

Living with diabetes involves regular appointments to check blood-sugar levels, blood pressure, etc. – how do you find these check-ups?

How do you find situations when health professionals ask probing questions about your lifestyle and diabetes?

Do you find that you are supported in relation to living with diabetes? By your family or treatment provider, for example?

Do you talk to your family, friends and/or colleagues about your diabetes?

If you have any questions about your diabetes, where do you search for answers?

If you have to tell others about diabetes, what do you say?

## Have you ever thought about potential long-term complications of your diabetes?

What do you think about cardiovascular screening invitations for people with diabetes?

Is there anything you would like to add or emphasise in relation to this conversation?

**Thank you very much for participating in our study**
